# Severity of *Giardia *infection associated with post-infectious fatigue and abdominal symptoms two years after

**DOI:** 10.1186/1471-2334-9-206

**Published:** 2009-12-15

**Authors:** Kristine Mørch, Kurt Hanevik, Guri Rortveit, Knut-Arne Wensaas, Geir Egil Eide, Trygve Hausken, Nina Langeland

**Affiliations:** 1 National Centre for Tropical Infectious Diseases, Haukeland University Hospital, Bergen, Norway; 2 Unit for Infectious Diseases, Department of Medicine, Haukeland University Hospital, Bergen, Norway; 3 Institute of Medicine, University of Bergen, Norway; 4 Department of Public Health and Primary Health Care, University of Bergen, Norway; 5 Research Unit for General Practice, Unifob Health, Bergen, Norway; 6 Centre for Clinical Research, Haukeland University Hospital, Bergen, Norway; 7 Unit for Gastroenterology, Department of Medicine, Haukeland University Hospital, Bergen, Norway

## Abstract

**Background:**

A high rate of post-infectious fatigue and abdominal symptoms two years after a waterborne outbreak of giardiasis in Bergen, Norway in 2004 has previously been reported. The aim of this report was to identify risk factors associated with such manifestations.

**Methods:**

All laboratory confirmed cases of giardiasis (n = 1262) during the outbreak in Bergen in 2004 received a postal questionnaire two years after. Degree of post-infectious abdominal symptoms and fatigue, as well as previous abdominal problems, was recorded. In the statistical analyses number of treatment courses, treatment refractory infection, delayed education and sick leave were used as indices of protracted and severe *Giardia *infection. Age, gender, previous abdominal problems and symptoms during infection were also analysed as possible risk factors. Simple and multiple ordinal logistic regression models were used for the analyses.

**Results:**

The response rate was 81% (1017/1262), 64% were women and median age was 31 years (range 3-93), compared to 61% women and 30 years (range 2-93) among all 1262 cases. Factors in multiple regression analysis significantly associated with abdominal symptoms two years after infection were: More than one treatment course, treatment refractory infection, delayed education, bloating and female gender. Abdominal problems prior to *Giardia *infection were not associated with post-infectious abdominal symptoms. More than one treatment course, delayed education, sick leave more than 2 weeks, and malaise at the time of infection, were significantly associated with fatigue in the multiple regression analysis, as were increasing age and previous abdominal problems.

**Conclusion:**

Protracted and severe *giardiasis *seemed to be a risk factor for post-infectious fatigue and abdominal symptoms two years after clearing the *Giardia *infection.

## Background

The intestinal protozoan *Giardia duodenalis *is a common cause of waterborne outbreaks in western countries since the cysts are relatively resistant to water treatment, and in most developing countries the parasite is highly endemic [1,2]. The clinical impact of *Giardia *infection ranges from mild self-limiting disease to life-threatening malnutrition [3-5].

Post-infectious complications have been described in children in developing countries, where giardiasis contributes to malnutrition and growth retardation with later risk for impaired cognitive function [6].

Also post-giardiasis complications in a western country have recently been described. We reported a high level of post-infectious fatigue (41%) and abdominal symptoms (38%) among 1017 cases two years after a waterborne outbreak in Bergen, Norway in 2004 [7,8]. The aim of this study was to investigate risk factors associated with such manifestations.

## Methods

### Study site and participants

Inclusion criteria was laboratory verified *Giardia *infection from the Bergen outbreak. All *Giardia*-positive cases diagnosed by stool microscopy (formalin-ether concentration) and/or antigen-test (ImmunoCard STAT! Cryptosporidium/*Giardia *rapid assay; Meridian Bioscience) at the local parasitology laboratory in Bergen were registered in the period from October 2004 to June 2005, and an additional 16 cases considered to be related to the outbreak were registered until December 2005. The majority of cases was registered in November 2004 (n = 863). A higher number than normal was registered also in 2005 (n = 172) after the water supply in December 2004 was considered free from *Giardia *cysts, probably due to protracted or secondary infections. A few cases known not to be infected in Bergen were excluded. According to this, a study population of 1262 cases, later referred to as "all cases", was defined.

The investigating laboratory is the only parasitology laboratory in the region, and prior to the outbreak about 50 cases a year were diagnosed, mainly infected abroad [9].

### Questionnaire

In August 2006, two years after the peak of the outbreak, a questionnaire was mailed to all cases, followed by an additional letter to non-respondents one month later.

We asked about abdominal problems prior to giardiasis; among these if abdominal problems had ever led to any of the following: Hospitalisation, sick leave, use of medication, seeing a doctor or problems without seeking health care, or if they did not have abdominal problems prior to September 2004.

Cases were asked about the following symptoms during giardiasis: Diarrhoea, abdominal pain, weight loss, malaise, nausea, foul smell and bloating. They were also asked about sick leave (0, <1, 1, 1-2 or >2 weeks), delayed education (0, <1, 1 or > 1 semester), time before abdominal symptoms disappeared (< 1, 1-3, 4-6 or 7-18 months) and number of treatment courses before abdominal symptoms ceased (0, 1, 2, >2, or recovered during treatment but relapsed).

We also asked if they had present problems with abdominal symptoms and with fatigue. The results for these latter questions have been published previously [8]. Present symptoms including nausea, bloating, abdominal pain, diarrhoea, constipation and reduced appetite were recorded on a scale from 0 (no symptoms) to 10 (severe symptoms). These questions have previously been used in recording severity of symptoms in patients with irritable bowel syndrome (IBS) [10].

Number of cases working/studying was estimated as cases answering the questions concerning sick leave/delayed education, minus cases answering that they were not working/studying.

Abdominal symptoms were reported as "no", "unsure" or "yes" on the question "Do you have abdominal symptoms now that you did not have prior to the *Giardia *infection?" Fatigue was reported as "less or same as usual", "more than usual" or "much more than usual" on the question "Do you have problems with fatigue?" These were used as ordinal response variables in the statistical analyses.

### Characteristics evaluated as possible risk factors

Whether protracted and severe *Giardia *infection could be associated with post-infectious fatigue or abdominal symptoms was studied, and number of treatment courses, delayed education and sick leave were all used as indices of protracted and severe infection in the statistical analyses. A sub-cohort of treatment resistant cases with mean symptom duration seven months, who had all been successfully treated in our out patient clinic, was also included in the statistical analyses. The characteristics and results from treatment of this cohort has been published previously [11]. Age, gender, previous abdominal problems and symptoms during infection were also evaluated as possible risk factors.

### Statistical methods

For each of the two ordinal response variables at two years follow-up, fatigue and abdominal symptoms, the possible risk factors were analysed as explanatory variables using ordinal logistic regression with the proportional odds model. In the proportional odds model the results are expressed as the ratio of the odds for a response above a certain level in the exposed to the odds for a response above this level in the unexposed. This odds ratio (OR) is modelled to be independent of the chosen level. First, for each of the response variables, simple ordinal logistic regression analysis was done for all risk factors. Then, all factors were included in a multiple ordinal logistic regression analysis (proportional odds model) [12]. From the analyses, unadjusted and adjusted odds ratios were estimated. To avoid loosing cases in the multiple regression analyses, missing values were included as valid categories in some variables as follows: "Not recovered" was included in the variable "Treatment courses", "Not working" in "Sick leave" and "Not a student" in "Delayed education". To evaluate the possible risk that these variables only reflect the response variables, the multiple regression analyses were performed with and without each of them. Age and gender were included into all models with no regards to significance. The final model included all variables with p < 0.05 (likelihood ratio test). Statistical analyses were performed using SPSS 15.0 or STATA.

### Ethics

Written informed consent was obtained. The regional ethics committee and the Privacy Ombudsman for Research approved the data collection and analyses of the study.

## Results

### Patient characteristics

Patient characteristics are shown in Table 1. The response rate was 81% (1017/1262), later referred to as "respondents". Median age among respondents was 31 years (range 3-93) and 64% were women, compared to 30 years (range 2-93) and 61% among all cases. Among the respondents, 722 (71%) were estimated to be working and 460 (45%) to be students. Prior to the *Giardia *outbreak, 12% of respondents (124/1017) had visited a doctor due to abdominal problems, 10% (98/1017) had had abdominal symptoms without seeking health care, 4% (42/1017) had used medication for such symptoms, 2% (19/1017) had been hospitalised and 2% (19/1017) had been on sick leave due to abdominal problems prior to the *Giardia *outbreak. A majority of 71% (718/1017) reported to have had no previous abdominal problems.

**Table 1:** Age and gender of the respondents and non-respondents to a postal questionnaire compared to all *Giardia*-positive cases during an outbreak in Bergen, Norway in 2004.

Characteristic	Respondents N (%)	Non-respondents N (%)	All cases N (%)
***Total***	1017 (100)	245 (100)	1262 (100)
***Gender***			
Female	647 (64)	117 (48)	764 (61)
Male	370 (36)	128 (52)	498 (39)
***Age ***(Years)			
0-19^a^	62 (6)	25 (10)	87 (7)
20-39	679 (67)	185 (76)	864 (68)
40-59	211 (21)	32 (13)	243 (19)
60-79	55 (5)	3 (1)	58 (5)
80-100	10 (1)	0 (0)	10 (1)

^a^Among respondents, 13 cases (1%) were less than 5 years, 13 cases (1%) were 5-9 years, 12 (1%) cases were 10-14 years and 24 (2%) cases were 15-19 years old.

Abdominal symptoms and fatigue two years after infection was recorded from 38% (389/1017) and 41% (419/1017) respectively as previously reported [8] (Table 2). Bloating was the symptom reported to be most severe (Table 2).

**Table 2:** Symptoms two years after *Giardia *infection in Bergen, Norway in 2004, among 1017 respondents to a questionnaire.

Symptoms	Cases^a ^(%)	Symptom score^b^	
		
		Mean ± SEM^c^	Median
***Fatigue***			
Less or same as usual	511 (50)		
More than usual	306 (30)		
Much more than usual	113 (11)		
***Abdominal symptoms***			
No	360 (35)		
Unsure	228 (22)		
Yes	389 (38)		
Bloating	475 (47)	4.57 ± 0.13	5.00
Diarrhoea	467 (46)	3.80 ± 0.12	3.74
Abdominal pain	442 (43)	3.64 ± 0.13	3.00
Nausea	416 (41)	2.23 ± 0.12	2.00
Reduced appetite	398 (39)	1.60 ± 0.12	0.67
Constipation	398 (39)	1.57 ± 0.12	0.66

^a ^Number of persons reporting the symptoms. Percent of respondents (n = 1017).

^b ^Symptoms scored from 0 (no symptoms) to 10 (severe symptoms).

^c ^Standard error of the mean.

### Giardia infection

Symptoms during *Giardia *infection, as reported two years later are shown in Table 3. Less than four weeks duration of symptoms, irrespective of treatment, was recorded in 18%. Only 1% became well without medication. Sick leave was reported by 46%, and almost half of these had been on sick leave for more than two weeks. Delayed education was reported in 33%, and 17% had one or more semester delay.

**Table 3:** Distribution of factors during *Giardia *infection among 1017 cases in Bergen, Norway in 2004, reported two years later.

Factors during *Giardia *infection	Cases (%)
***Symptoms***	
Diarrhoea	904 (89)
Malaise	761 (75)
Abdominal pain	689 (68)
Bloating	657 (65)
Nausea	647 (64)
Foul smell	612 (60)
Weight loss	539 (53)
None of these symptoms	15 (2)
***Duration of abdominal symptoms***	
< 4 weeks	185 (18)
1-3 months	193 (19)
4-6 months	88 (9)
7-18 months	62 (6)
***Number of treatment courses***	
0	13 (1)
1	348 (34)
2 or more	153 (15)
Recovered during treatment but relapsed	84 (8)
***Duration of sick leave *(n = 722)^a^**	
No sick leave	394 (54)
< 1 week	62 (9)
1-2 weeks	110 (15)
> 2 weeks	157 (22)
***Delayed education *(n = 460)^b^**	
No delay	311 (68)
< 1 semester	69 (15)
1 semester	44 (10)
> 1 semester	36 (8)

^a ^Percent of persons estimated to be working (n = 722).

^b ^Percent of persons estimated to be students (n = 460).

### Factors associated with fatigue and abdominal symptoms

The analyses of risk factors associated with abdominal symptoms and fatigue are presented in Table 4 and 5, respectively.

**Table 4:** Abdominal symptoms two years after *Giardia *infection reported in a cohort of 1017 cases, associated with patient characteristics and factors during giardiasis.

Characteristics	N	Abdominal symptoms							
		
		No N (%)	Unsure N (%)	Yes N (%)	Simple regression analysis^a^		Multiple regression analysis^b^		
					
					OR	p-value	OR	p-value	95% CI
**Gender**								0.029	
Male	355	138 (39)	87 (25)	130 (37)	1.00		1.00		
Female	622	222 (36)	141(23)	259 (42)	1.20	0.158	1.45		(1.04, 2.04)
									
**Age^c ^(years)**	977				1.01	0.064	-	Ns	-
									
**Abdominal problems before 2004 leading to**									
Visiting doctor	119	35 (29)	39 (33)	45 (38)	1.13	0.516	-	Ns	-
Hospitalisation	17	4 (24)	8 (48)	5 (30)	1.04	0 925	-	Ns	-
Sick leave	18	7 (39)	8 (44)	3 (17)	0.62	0.286	-	Ns	-
Use of medication	40	12 (30)	16 (40)	12 (30)	0.94	0.826		Ns	
Problems without seeking health care	94	30 (32)	26 (28)	38 (40)	1.13	0.539	-	Ns	-
**No abdominal problems before 2004**	883	330 (37)	202 (23)	351 (40)	1.18	0.220	-	Ns	
									
**Acute giardiasis**									
Diarrhoea	880	318 (36)	211 (24)	351 (40)	1.19	0.390	-	Ns	-
Abdominal pain	674	212 (32)	160 (24)	302 (45)	2.05	<0.001	-	Ns	-
Weight loss	529	187 (35)	128 (24)	214 (41)	1.10	0.411	-	Ns	-
Malaise	742	242 (33)	183 (25)	317 (43)	1.91	<0.001	-	Ns	-
Nausea	632	226 (36)	150 (24)	256 (41)	1.11	0.391	-	Ns	-
Foul smell	595	178 (30)	146 (25)	271 (46)	2.00	<0.001	-	Ns	
Bloating	645	176 (27)	158 (25)	311 (48)	3.20	<0.001	1.51	0.023	(1.06, 2.16)
									
**Treatment refractory infection^d^**	28	7 (25)	6 (21)	15 (54)	1.78	0.117	3.08	0.030	(1.11, 8.53)
									
**Treatment courses^e^**								<0.001	
0 or 1	360	242 (67)	85 (24)	33 (9)	1.00		1.00		
2 or more	237	99 (42)	69 (29)	69 (29)	3.23	<0.001	3.39	<0.001	(2.33, 4.94)
Recovered during course but relapsed	3	1 (33)	1 (33)	1 (33)	4.38	0.166	5.05	Ns	(0.57, 45.11)
Not recovered	374	16 (4)	73 (20)	285 (76)	30.54	<0.001	32.81	<0.001	(21.67, 49.65)
									
**Sick leave^e^**								Ns	
No sick leave	387	162 (42)	85 (22)	140 (36)	1.00		1.00		
< 1 week	61	20 (33)	19 (31)	22 (36)	1.21	0.460	-	Ns	-
1-2 weeks	105	41 (39)	32 (31)	32 (31)	0.95	0.806	-	Ns	-
> 2 weeks	153	43 (28)	27 (18)	83 (54)	2.04	<0.001	-	Ns	-
Not working	241	85 (35)	60 (25)	96 (40)	1.26	0.150	-	Ns	-
									
**Delayed education^e^**								0.020	
No delay	306	140 (46)	65 (21)	101 (33)	1.00		1.00		
< 1 semester	67	25 (37)	15 (22)	27 (40)	1.40	0.175	1.06	Ns	(0.60, 1.89)
1 semester	42	9 (21)	10 (24)	23 (55)	2.69	0.002	2.47	0.024	(1.13, 5.45)
> 1 semester	34	1 (3)	8 (24)	25 (74)	6.64	<0.001	3.50	0.013	(1.30, 9.38)
Not a student	379	141 (37)	91 (24)	147 (39)	1.36	0.031	3.14	Ns	(0.80, 1.63)

Abbreviations: OR, odds ratio; Ns, not significant; CI, confidence interval.

^a ^Simple ordinal logistic regression analysis (proportional odds model).

^b ^Ordinal logistic regression analysis. Multiple regression analysis included all variables in the table. Missing values; n = 205.

^c ^Age as continuous variable in the statistical analysis.

^d ^All cases successfully treated with albendazole + metronidazole, paromomycin or quinacrine + metronidazole after 30 (mean) weeks duration of infection [11].

^e ^Categorical variable in the statistical analysis including the categories shown in the table. Likelihood-ratio test (STATA).

**Table 5:** Fatigue two years after *Giardia *infection reported in a cohort of 1017 cases, associated with patient characteristics and factors during giardiasis.

Characteristics	N	Fatigue							
		
		Not more than usual N (%)	More than usual N (%)	Much more than usual N (%)	Simple regression analysis^a^		Multiple regression analysis^b^		
					
					OR	P-value	OR	p-value	95% CI
**Gender**								Ns	
Male	338	199 (59)	107 (32)	32 (10)	1.00		1.00		
Female	592	312 (53)	199 (34)	81 (14)	1.32	0.038	-		
									
**Age^c ^(years)**	930				1.02	<0.001	1.02	0.007	(1.01, 1.03)
									
**Abdominal problems before 2004 leading to**									
Visiting doctor	114	49 (43)	50 (44)	15 (13)	1.56	0.020	-	Ns	
Hospitalisation	14	4 (29)	8 (57)	2 (14)	2.21	0.113	-	Ns	
Sick leave	17	6 (35)	10 (59)	1 (6)	1.60	0.309	-	Ns	
Use of medication	35	15 (43)	18 (51)	2 (6)	1.31	0.414	-	Ns	
Problems without seeking health care	95	45 (47)	40 (42)	10 (11)	1.26	0.256	2.13	0.025	(1.10, 4.15)
**No abdominal Problems before 2004**	662	380 (57)	198 (30)	84 (13)	0.78	0.081	-	Ns	
									
**Acute giardiasis**									
Diarrhoea	839	456 (54)	279 (33)	104 (12)	1.28	0.255	-	Ns	
Abdominal pain	646	322 (50)	236 (37)	88 (14)	1.95	<0.001	-	Ns	
Weight loss	506	255 (50)	180 (36)	71 (14)	1.50	0.002	-	Ns	
Malaise	714	334 (47)	277 (39)	103 (14)	5.03	<0.001	3.68	<0.001	(2.25, 6.01)
Nausea	604	302 (50)	215 (36)	87 (14)	1.81	<0.001	-	Ns	
Foul smell	580	290 (50)	201 (35)	89 (15)	1.80	<0.001	-	Ns	
Bloating	622	311 (50)	224 (36)	87 (14)	1.84	<0.001	-	Ns	
									
**Treatment refractory infection^d^**	29	13 (45)	11 (38)	5 (17)	1.52	0.236	-	Ns	
									
**Treatment courses^e^**								<0.001	
0 or 1	326	249 (76)	68 (21)	9 (3)	1.00		1.00		
2 or more	227	128 (56)	77 (34)	22 (10)	2.55	<0.001	1.98	0.002	(1.28, 3.07)
Recovered during course but relapsed	3	2 (67)	1 (33)	0 (0)	1.50	0.742	2.37	Ns	(0.19, 29.08)
Not recovered	371	129 (35)	160 (43)	82 (22)	6.39	<0.001	6.20	<0.001	(4.16, 9.24)
									
**Sick leave^e^**								0.001	
No sick leave	368	232 (63)	106 (29)	30 (8)	1.00		1.00		
< 1 week	58	30 (52)	24 (41)	4 (7)	1.45	0.180	0.93	Ns	(0.49, 1.77)
1-2 weeks	98	63 (64)	29 (30)	6 (6)	0.93	0.740	0.72	Ns	(0.42, 1.23)
> 2 weeks	150	55 (37)	50 (33)	45 (30)	3.63	<0.001	2.26	<0.001	(1.45, 3.53)
Not working	232	125 (54)	84 (36)	23 (10)	1.42	0.035	1.08	Ns	(0.71, 1.63)
									
**Delayed education^e^**								0.001	
No delay	291	205 (70)	67 (23)	19 (7)	1.00		1.00		
< 1 semester	66	31 (47)	30 (46)	5 (8)	2.34	0.002	1.79	0.049	(1.00, 3.19)
1 semester	39	13 (33)	17 (44)	9 (23)	4.64	<0.001	2.80	0.004	(1.39, 5.66)
> 1 semester	35	8 (23)	13 (37)	14 (40)	9.21	<0.001	3.88	<0.001	(1.82, 8.30)
Not a student	357	188 (53)	121 (34)	48 (13)	2.17	<0.001	1.51	0.033	(1.03, 2.21)

Abbreviations: OR, odds ratio; Ns, not significant.

^a ^Simple ordinal logistic regression analysis (proportional odds model).

^b ^Ordinal logistic regression analysis (proportional odds model). Multiple regression analysis included all variables in the table. Missing values; n = 241

^c ^Age as continuous variable in the statistical analysis.

^d ^All cases successfully treated with albendazole + metronidazole, paromomycin or quinacrine + metronidazole after 30 (mean) weeks duration of infection [11].

^e ^Categorical variable in the statistical analysis including the categories shown in the table. Likelihood-ratio test (STATA)

More than one course of treatment for giardiasis, as well as delayed education, was significantly associated with both fatigue and abdominal symptoms in all analyses. Previous abdominal problems were not significantly associated with post-infectious abdominal symptoms in any analyses.

Female gender turned out not to be significantly associated with abdominal symptoms when the variable "treatment courses" was left out from the multiple regression analysis (data not shown).

## Discussion

We have previously reported a high prevalence of fatigue (41%, 419/1017) and abdominal symptoms (38%, 389/1017), and a highly significant association between these symptoms, two years after the Bergen outbreak [8]. In this study we report an association between protracted and severe *Giardia *infection and such symptoms.

Fatigue has been described following Q-fever, *Epstein Barr *virus infection, *Ross River *virus infection, brucellosis, Lyme disease, viral meningitis and Dengue fever, and in the first three of these, severe infection was a risk factor [13-16].

In a sub-cohort of referred cases after the Bergen outbreak, 81% (66/82) fulfilled the Rome II criteria for IBS [17]. Post-infectious (PI)-IBS has been reported with a median prevalence of around 10% following gastroenteritis of various aetiology; *Campylobacter *[18-20], *Salmonella *[21-23], *Shigella *[24-26], *Escherichia coli *[20] and *Trichinella spiralis *[27], and severe infection was the most important risk factor in these reports [24,28-30].

A possible weakness in our definition of indices of protracted and severe *Giardia *infection is that sick leave or delayed education may be signs of PI-IBS or fatigue rather than protracted infection or inflammation. Particularly those reporting ">1 semester delayed education", but also "sick leave > 2 weeks", could potentially be cases actually reporting persistent symptoms, but excluding them from the statistical analyses would have created a selection bias. More than one treatment course is a reliable index of persistent infection and the finding of this as a highly significant risk factor in all analyses supports the hypothesis that protracted and severe infection may cause post-infectious complications. However, some patients may not have had stool samples tested between the courses, and may therefore have experienced post-infectious complaints rather than protracted infection. Results regarding the sub-cohort of referred treatment refractory cases must be interpreted with caution due to the very low sample size (n = 29) [11].

If our findings are causal, then the surprisingly high level of fatigue and abdominal symptoms reported, may have two possible explanations: A substantial number of cases had protracted infection due to delayed treatment explained by late detection of the outbreak [7], and/or the infection caused inflammation in a high number of cases, also after clearing the parasites [31]. Giardiasis may cause damage to the intestinal epithelial brush border, villous atrophy and inflammation [32]. In duodenal biopsies from a sub-cohort of 124 cases with symptoms for 8 months (mean) from the Bergen outbreak, inflammation was found in 47% [31], which is surprisingly high compared to a previous report of duodenal inflammation in 4% of 462 *Giardia *cases from Germany [33]. This supports that protracted *Giardia *infection, and possibly protracted intestinal inflammation, may have induced PI-IBS and fatigue in this cohort. Low-grade inflammation in gut-mucosa has been described as a possible mechanism for IBS also following other infections [34].

The high prevalence of inflammation in this outbreak may be explained by immunological host factors, but probably also by parasite virulence factors. Studies addressing the possible association between *Giardia *genotypes and clinical manifestations have shown different results: Both genotype A and B have been responsible for severe symptoms, with the genotype less prevalent in the community responsible for more severe clinical manifestation [35-38]. In Norway 11% of raw water samples contains *Giardia *parasites in low concentrations, most commonly genotype A, while the outbreak strain in Bergen was assemblage B [39,40]. Previous studies from the outbreak suggest that some sub-genotypes induced a more severe infection [11,41], and one may speculate if particular sub-genotypes lead to an immune reaction responsible for protracted low-grade inflammation. Also in IBS following infections other than giardiasis, toxicity within species has been reported as a risk factor, as in *Campylobacter *infection [18].

Age was significantly associated with fatigue as previously reported from this cohort [8], which is in line with findings in a Norwegian population study [42]. We did not find any significant association between age and abdominal symptoms. Age >60 years was protective against PI-IBS in one study [29], and one mechanism was suggested to be that the gut in older subjects might be less reactive to infection [43].

Female gender seemed to be a risk factor for both fatigue and abdominal symptoms in some but not all analyses, and this finding must therefore be interpreted with caution. An association between chronic fatigue and female gender has been reported in other studies [13,42,44-46]. Female gender has also been associated with PI-IBS in some reports; however, this association was no longer significant when analyses were adjusted for psychological disturbances [19,28,29,47].

Malaise during infection was associated with fatigue, and bloating with abdominal symptoms. This may either indicate recall bias influenced by symptoms present at the time of reporting, or a real risk of complications if these symptoms are prominent.

There was no association between previous abdominal problems and post-infectious abdominal symptoms. The finding that "previous abdominal problems without seeking health care" was associated with fatigue should be interpreted with caution since other factors measuring previous abdominal problems were not associated. The possible impact of recall bias on the outcome regarding previous abdominal problems in this study may be less since the questions were phrased as facts ("hospitalised" etc) rather than symptoms.

Chronic *Giardia *infection may develop in 15% of patients if not treated [4], and may be misdiagnosed as IBS due to similar symptoms [48]. *Giardia *was found in 6.5% of IBS cases in one study from Italy [49], and in a meta-analysis from the Nordic countries, giardiasis was found in 3.0% of the asymptomatic, and in 5.8% of the symptomatic, population [50].

We have previously shown that all referred treatment refractory cases after the Bergen outbreak eradicated the parasite, evaluated by seven negative stool samples up to four weeks after treatment [11]. In 25 *Giardia *negative patients from this outbreak, treatment with metronidazole/albendazole or tetracycline/folic acid was not effective, and cryptic giardiasis was therefore excluded [51]. We have also described that IBS persisted in patients after normalisation of duodenal biopsies [17]. These reports support that chronic infection is not the cause of fatigue and abdominal symptoms in this cohort.

A major strength of the study is the large number of cases, and the high response rate. A limitation may be the risk of over-reporting of symptoms since local authorities have accepted to compensate any economical loss due to the infection. Possible reporting bias due to limitations in the questionnaire could have been reduced if validated measures of chronic fatigue and IBS had been used in a controlled study. Also, the study lacks information on co-morbidity and professional background. It is unclear whether these limitations have influenced the outcome of the study. On the other hand, the study was initiated by clinicians after observing a substantial number of patients with severe chronic fatigue and/or abdominal symptoms after giardiasis, supporting that self reporting of symptoms associated with giardiasis in this cohort is consistent with clinical observations.

## Conclusions

Protracted and severe *Giardia *infection, or possibly inflammation, seems to be a risk factor for post-infectious fatigue and abdominal symptoms two years after clearing the parasites. These symptoms are often pronounced, reduce quality of life and have economical implications in the society. If the observed association is causal, shortening the duration of *Giardia *infection by early diagnosis and treatment may be important also in order to reduce the risk for such complications.

## Competing interests

The authors declare that they have no competing interests.

## Authors' contributions

KM, KH, GR, KAW, TH and NL participated in the design of the study. KM collected data and drafted the manuscript. GEE and KM performed the statistical analyses. All authors contributed to interpretation of data, revision of the manuscript, and approved the final manuscript.

## Pre-publication history

The pre-publication history for this paper can be accessed here:

http://www.biomedcentral.com/1471-2334/9/206/prepub
